# New York Pathological Society

**Published:** 1868-02

**Authors:** B. H. Sands

**Affiliations:** President, in the Chair


### NEW YORK PATHOLOGICAL SOCIETY.

### Stated Meeting, November 27, 1867.

Dr. B. H. SANDS, President, in the Chair.

### VALUABLE EXPERIMENTS ON LIGATION OF ARTERIES.

Dr. Benj. Howard presented a series of specimens, stating, that as he had six recent cases to exhibit, he would, in description, confine himself to the chief points in the respective cases, and would endeavor to avoid a repetition of anything expressed upon the same subject upon a previous occasion.

The history of the first specimen I present, marked No. 5, is as follows: Desirous of observing the result of a simple apposition of the internal coats of an artery, I prepared a band of lead, about a line in width, smoothed all sharp edges, and polished it brightly; its length was such that, when doubled upon itself, it sufficiently exceeded the diameter of the artery as to allow of its being passed around the vessel, and the two ends clamped together, without any mechanical injury to the coats of the vessel. I applied this ligature in the manner described, to the right common carotid artery of a sheep, on the 17th of October. On the 23d of November, being thirty-seven days after the operation, I made an incision in the line of the cicatrix, which was well healed, when I found, close beneath the surface, the ligature in a state of dryness, and waiting to drop from the cicatrix. The deposit of fibrine is seen to have been quite extensive, forming a solid mass from the artery to the integument. Through the whole extent of this will be observed a sinus communicating with the place where the ligature was found, and the point of its application.

The longitudinal section of the artery shows that the occlusion of the artery by fibrine at the point of ligation, and by a well-formed plug above and below it, is perfect. The ligature appears to contain in its embrace, the part of the artery it was made to include.

No. 6.—October 17. I applied to the right common carotid of another sheep, a lead-wire ligature, tying it loosely, diminishing calibre about two-thirds. November 22d, being thirty-six days after, I found this cicatrix healed, but at about its middle, a small fluctuating tumor. On making an incision through it along the line of cicatrix, the tumor was found to contain about a drachm of very thick pus, in which I found the ligature. The deposit of fibrine will be seen to be greater in this than in the last specimen; and through its whole extent, from its point of application to the abscess, the ligature had ulcerated its way. It is very interesting to observe the plug of fibrine, which is clearly shown *in situ*, by which the whole rear track of the ligature has been closed up as it advanced toward the integument.

The longitudinal section shows, as do others, that at the point of ligation, for the portion of artery destroyed by the ligature, is substituted a solid mass of fibrine, and that the artery is thus perfectly occluded, as well as by the clot above and below.

No. 10.—October 31. To the right carotid of a sheep, I applied a flat silk ligature outside its sheath, diminishing the calibre of the artery barely one-half. November 23d, being twenty-three days after, I found the cicatrix soundly healed, the fibrous deposit smaller than in either of the two preceding cases; and you will observe that the longitudinal section of the artery reveals the same dense fibrine substituted for the canal of the artery at the point of ligation.

There is no sinus or trace of the ligature visible in this specimen, as there was in the other cases. But we know it did escape, because it was found outside the wound, the free end having been fastened there at the time of ligation.

No. 7. My object in the case now before us, was to observe the effect upon an artery of a simple cessation of function.

Accordingly, October 31st, I applied three silver-wire ligatures, the second being about twelve lines above the first, and the third about four lines above the second.

The first was tied with little less than the average tightness commonly used in the silk ligature; the second broke close to the loop, so that I had but to cut off the free end; the third I tied with firmness, but with more caution, to avoid repetition of the accident incident to the second; twenty-two days after, I vivisected this specimen, the cicatrix was not firm and the hemorrhage was great. It will be seen that the calibre of the arterial canal, between the ligatures, is very much diminished, and is occupied towards its centre by what appears to be an organized clot, which, as we approach the points of ligation on either hand, becomes a firm plug of fibrine.

The history of the disused artery is, however, of but small importance, compared with that of the ligatures as here observed. The first ligature cannot be found. The second is also wanting; and from the original site of each throughout the fibrinous mass, toward the cicatrix, you will observe an open sinus through which they have been discharged. The third ligature, applied with less force, will be seen lying loosely within the artery, at the internal end of the upper sinus, apparently waiting to be extruded. The occlusion about each ligated point is complete.

No. 9. This is the right carotid of a sheep, which I ligated October 31. The ligature was of silver wire, the same as that used in the last specimen exhibited. I tied it by a single flat knot, and cut off the ends closely. I applied it outside the sheath, tying loosely, and diminishing the calibre of the artery about half or two-thirds.

November 23d, being twenty-three days after the operation, I found the cicatrix quite sound. The vivisection was accompanied with very little hemorrhage, and here is the specimen. There is but little deposition of fibrine, and a longitudinal section of the artery reveals the ligature *in situ*, as applied; the looseness of its application being manifest by the size of the piece of whalebone passed through its loop. It will be seen there is no sign of suppuration or sloughing, but only the deposition of fibrine for the envelopment of the ligature, and that due to inflammation following the laceration of the areolar tissue in the performance of the operation.

Here is a specimen exhibiting the difference between the effect of the "surgeon's" or flat knot, and the ordinary knot, upon the internal and middle coats.

In neither case are the divided edges of the inner coats in apposition; in the former case you will observe, by means of the lens which I will pass around with it, that the divided ends are curled upward, their flat surfaces forming a plane representing the diameter of the artery, at right angles with its long axis; they are therefore not in apposition with each other, but only in juxtaposition.

The ordinary round knot, you will see, has produced a puckering and partial laceration of the inner coats, the plications terminating like radii in the centre of the transverse section exhibited.

*The specimens presented exhibit in each case where a loose ligature has been used, whether of silver, lead, or silk, perfect occlusion of the arterial canal.*

*When the loose ligature has consisted of lead or of silk, ulceration and suppuration have occurred, with extrusion of the ligature toward the surface.*

Equally, with other specimens previously presented, these exhibit that *every* TIGHT *ligature of either kind, and every* LOOSE *ligature except the* SILVER, *has been extruded; but the "loose silver ligature" is followed by obliteration, fibrinous and extensive, with exemption from sloughing, from suppuration, and from extrusion of ligature common to every case of other kinds exhibited.*

Here are the facts, as far as they go. The theory which after a sufficient accumulation may be woven out of them, may be worthy the consideration of the Society. As to the causes of the differences in results, I may be allowed to suggest that the questions in order might be:

1st.—*The Ligature.* What relation exists between the different qualities of the different ligatures, and the difference in the results exhibited respectively?

2d.—*Looseness.* In the absence of sudden lesion of the artery, and of sudden complete strangulation of the vasa vasorum, are not the parts better able to perform the new task suggested and assigned to them?

Is not the operation, the shock, the local and general disturbance more moderate than in the case of a tight ligature?

Is not the gradual accommodation of the collateral branches to the demand instituted on the application of a loose ligature, sufficient to account for the absence of the great oozing common only to all the vivisections made in the region of a recent *tight* ligation?—*Medical Record.*

